# Computational Fluid Dynamics (CFD) as a Tool for Investigating Self-Organized Ascending Bubble-Driven Flow Patterns in Champagne Glasses

**DOI:** 10.3390/foods9080972

**Published:** 2020-07-23

**Authors:** Fabien Beaumont, Gérard Liger-Belair, Guillaume Polidori

**Affiliations:** 1 Physique et Sciences Pour l’Ingénieur (PSPI), Université de Reims Champagne-Ardenne, UFR Sciences Exactes et Naturelles, BP 1039, CEDEX 2, 51687 Reims, France; fabien.beaumont@univ-reims.fr (F.B.); guillaume.polidori@univ-reims.fr (G.P.); 2 Equipe Effervescence, Champagne et Applications (GSMA-UMR CNRS 7331), Université de Reims Champagne-Ardenne, UFR Sciences Exactes et Naturelles, BP 1039, CEDEX 2, 51687 Reims, France

**Keywords:** champagne, bubbles, flow patterns, CFD, PIV, VOF method

## Abstract

Champagne glasses are subjected to complex ascending bubble-driven flow patterns, which are believed to enhance the release of volatile organic compounds in the headspace above the glasses. Based on the Eulerian–Lagrangian approach, computational fluid dynamics (CFD) was used in order to examine how a column of ascending bubbles nucleated at the bottom of a classical champagne glass can drive self-organized flow patterns in the champagne bulk and at the air/champagne interface. Firstly, results from two-dimensional (2D) axisymmetric simulations were compared with a set of experimental data conducted through particle image velocimetry (PIV). Secondly, a three-dimensional (3D) model was developed by using the conventional volume-of-fluid (VOF) multiphase method to resolve the interface between the mixture’s phases (wine–air). In complete accordance with several experimental observations conducted through laser tomography and PIV techniques, CFD revealed a very complex flow composed of surface eddies interacting with a toroidal flow that develops around the ascending bubble column.

## 1. Introduction

Champagne and sparkling wines elaborated through the same traditional method are under a high pressure of carbon dioxide (CO_2_), because gas-phase CO_2_ forms together with ethanol during a second in-bottle fermentation process promoted by adding yeasts and a certain amount of sugar in bottles hermetically sealed with a crown cap or a cork stopper [1]. This second in-bottle fermentation process forces an amount equivalent to around 11–12 g L^−1^ of CO_2_ to progressively dissolve into the wine, according to so-called Henry’s law [1]. Immediately after uncorking a bottle of champagne, the thermodynamic equilibrium between gas-phase and dissolved CO_2_ is broken, and the liquid phase becomes supersaturated with CO_2_. Dissolved CO_2_ must therefore progressively desorb from the liquid phase. As firstly observed by Liger-Belair et al. [2], massive losses of dissolved CO_2_ are indeed experienced by champagne during the pouring step. Immediately after pouring champagne into a glass, the dissolved CO_2_ concentration falls to a level in the order of 6–9 g L^−1^, depending on several parameters, such as the champagne temperature, the bottle type, or the glass shape, for example [2,3,4,5]. This range of dissolved CO_2_ concentration of 6–9 g L^−1^ is nevertheless well beyond the required minimum level of dissolved CO_2_, close to 2.5 g L^−1^, to enable heterogeneous bubble nucleation under standard tasting conditions [6].

Under standard tasting conditions, there are two main pathways for the release of dissolved CO_2_ from champagne and sparkling wine glasses: (i) into the form of heterogeneously nucleated bubbles (the so-called effervescence process) and (ii) through the molecular diffusion of dissolved CO_2_ through the air/wine interface (indeed invisible by the naked eye), as described in minute detail by Liger-Belair [6]. In recent years, glassmakers proposed to champagne and beer-drinkers a new generation of laser-etched glasses specially designed to cause the standardized conditions of effervescence [7]. In laser-etched glasses, bubble nucleation is most often triggered at the bottom of the glass with a ring-shaped structure made with adjoining laser beam impacts. Laser-etched glasses filled with champagne, beer, or any other sparkling beverage, are thus easily recognizable, with a continuously renewed bubbly flow ascending along their vertical axis of symmetry. A bubbling scenario from such laser-etched champagne glasses was indeed recently proposed [7].

Actually, due to viscous effects, buoyant bubbles triggered and constantly released from the bottom of laser-etched glasses create a field of adverse pressure in their near-wakes as they rise towards the free surface. In laser-etched champagne glasses, the champagne bulk is therefore subjected to a continuous central bubbly flow, resulting in a very efficient stirring process [8] (as long as the champagne holds enough dissolved CO_2_ to promote heterogeneous bubble nucleation [6]). The homogeneous stirring of champagne under the action of rising bubbles confers an advantage compared with a situation where the liquid phase is at rest by renewing the air/champagne subsurface layers with champagne from the bulk, thus enhancing the release of dissolved CO_2_, as well as the evaporation of volatile organic compounds (VOCs) in the headspace above the glass [9,10].

In light of all of this, a large body of research has been developed in the past 15 years to investigate the interplay between ascending bubbles, glass shape and flow patterns, and their combined action on the overall sensation perceived by champagne tasters. Laser tomography coupled with particle image velocimetry (PIV) techniques were used in order to visualize the three-dimensional (3D) flow patterns forced by an ascending bubbly flow in the bulk of various laser-etched champagne glasses [11,12,13,14,15,16,17]. Recently, infrared thermography imaging was also coupled with PIV to unveil the close interplay between flow patterns found in champagne glasses and the release of gas-phase CO_2_ from the champagne surface [18]. Computational fluid dynamics (CFD) methods have also been applied to model ascending bubble-driven flow patterns in champagne glasses [19,20,21]. In stout beer glasses (such as the Guinness beer glass), a combined CFD and experimental approach was performed to demonstrate that the bubbly flow also strongly depends on the shape of the glass [22]. If it narrows downwards, as the traditional stout glass does, the flow is directed downwards near the wall and upwards in the interior (and sinking bubbles are observed), whereas, if the container widens downwards, the flow is opposite to that described above and only rising bubbles can be seen. More recently, a Japanese team has revisited this visually appealing phenomenon observed in glasses filled with this famous Irish stout beer [23]. By combining direct observations with laser-induced fluorescence and computer models, the researchers created a detailed profile of the liquid phase velocity. They found that the bubbles-going-down effect, observed with bubbles much smaller than those found in most standard beers or champagnes, is analogous to roll waves commonly observed in water sliding downhill on a rainy day [23].

In the present article, based on the Eulerian–Lagrangian approach, CFD was used in order to examine how a column of ascending bubbles nucleated at the bottom of a classical champagne glass can drive self-organized flow patterns, both in the champagne bulk and at the air/champagne interface. Previous sets of flow visualization data unveiled through laser tomography and PIV techniques in the past will thus be compared with 2D and 3D CFD models to further explore how ascending bubble-driven flow patterns develop and evolve over time in a laser-etched champagne glass, under standard tasting conditions.

## 2. Modeling the Glass and Physicochemical Parameters of Champagne

### 2.1. Modeling the Champagne Glass

**Figure 1:**
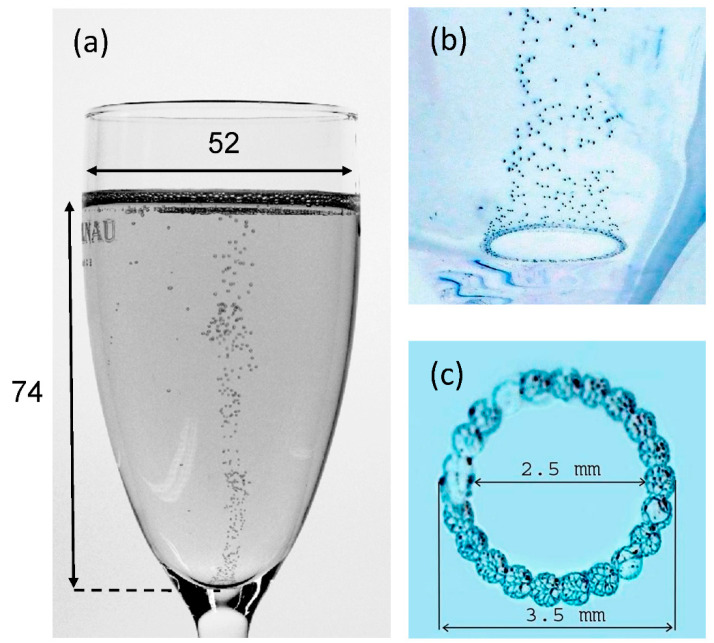
Photographic evidence for bubble production from the ring-shaped structure made at the bottom of a laser-etched flute filled with 100 mL of champagne [7] (**a**). Detail of the ring-shaped structure made with adjoining laser beam points of impact (**b**,**c**). In frame (**a**), dimensions are indicated in mm.

In this study, a tulip-shaped flute classically used for champagne and sparkling wine tasting [7] was modeled for the 2D CFD approach. The flute was laser-etched at its bottom in order to promote a standardized bubbly flow ascending along its axis of symmetry, as shown in the photographs displayed in Figure 1.

**Figure 2:**
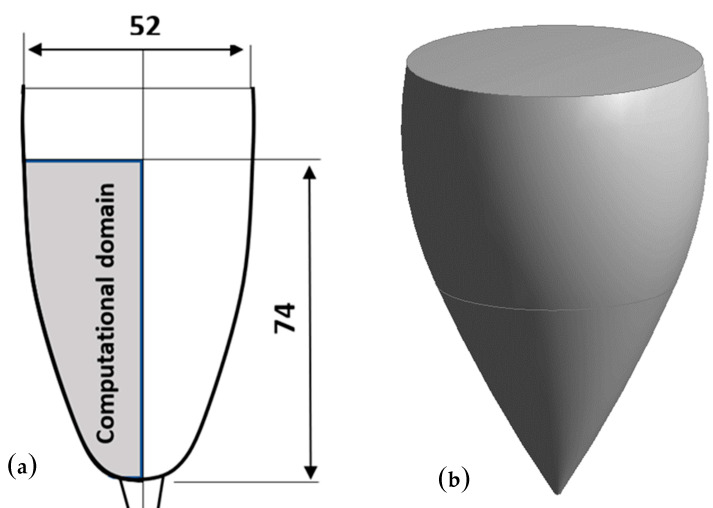
Computational domain, in gray, as determined for the 2D computational fluid dynamics (CFD) approach (**a**) and a 3D sketch of the glass, as determined for the 3D CFD approach (**b**). In frame (**a**), dimensions are indicated in mm for the glass filled with 100 mL of champagne.

In previous works, laser tomography techniques revealed the axisymmetric behavior of the swirling flow found in such laser-etched champagne glasses [11,12,13,14,15,16,17]. Hence, a 2D axisymmetric model can be used and only half of the fluid domain can be considered, thus strongly limiting the computation cost. The computational domain defined for the 2D CFD approach is shown in Figure 2a, whereas the 3D sketch of the glass, as determined for the 3D CFD approach, is shown in Figure 2b.

The 3D computer-aided geometric design had dimensions equivalent to those of the 2D model. The 3D calculation area had a total height of 76 mm (with a level of champagne of 74 mm and a 2 mm thick buffer zone filled with air above the champagne surface). In our numerical procedure, we simulated the emergence of CO_2_ bubbles into the form of a bubbly flow (equivalent to the bubbles heterogeneously nucleated from the laser etchings presented in Figure 1) through injection points found at the bottom of the glass.

### 2.2. Meshing of the Computational Domain

**Figure 3:**
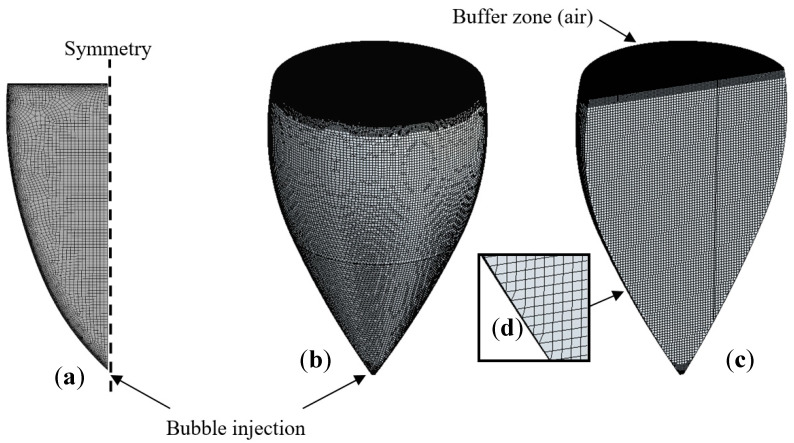
The 2D meshing of the fluid domain (**a**) and 3D meshing of the fluid domain (**b**), respectively. The 2D meshing as seen in the plane of symmetry of the glass (**c**) and detail of the mesh close to the glass wall (**d**).

The discretization of the calculation domains (2D and 3D) was achieved using ANSYS^®^ Workbench Meshing software. Because 2D modeling is carried out with axisymmetric calculations, only half of the fluid domain is meshed, as seen in Figure 3. The 2D mesh is composed of about 7700 quadrilateral and hexahedral elements, with a structured grid in the central part of the computational domain and an unstructured grid in the vicinity of the glass wall. To ensure that the results of the CFD calculation were consistent with the experimental results, a grid dependency test was performed. The convergence test consisted of improving the results by using successively smaller cell sizes. More details about the grid dependency test can be found in a previous study [19].

The 3D mesh is composed of about 747,500 hexahedral elements refined near the air/wine interface and at the bottom of the glass where the bubbles are injected. In order to correctly model the dynamics of ascending bubbles along their vertical journey toward the champagne surface, the dimensions of mesh elements were chosen according to the continuous increase in ascending bubbles’ diameters, ensuring that the mesh size remains smaller than the bubble size. It is worth noting that, under real champagne tasting conditions, ascending bubbles leave their nucleation site with a diameter close to 50 μm at the bottom of the flute and grow to a diameter close to 1 mm when they hit the free surface of the liquid (as they follow an upward journey of several cm). In the same way as for the 2D model, a grid dependency test was carried out for the 3D model [21].

### 2.3. Physicochemical Parameters of Champagne and Gas-Phase CO_2_

Some classical physicochemical parameters of a standard commercial Champagne wine (with 12.5% *v/v* of ethanol) have already been determined in a previous work, at 20 °C, with samples of champagne first degassed under vacuum [24]. Its static surface tension *γ* was found to be in the order of 50 mN m^−1^, its density *ρ* was found to be very close to that of water (i.e., 10^−3^ kg m^−3^) and its dynamic viscosity was found to be about 50% higher than that of pure water (mainly because of ethanol). The initial concentration of dissolved CO_2_ (denoted [CO_2_]_ini_) found in the liquid phase immediately after pouring 100 mL of champagne into the specific glass described above was also determined in a previous work [4]. Moreover, the diffusion coefficient of molecular species in a liquid phase, such as dissolved CO_2_ molecules in champagne, for example, is therefore strongly temperature dependent. The diffusion coefficients of dissolved CO_2_ molecules in a standard commercial Champagne wine (denoted *D*) was accurately determined as a function of temperature by classical molecular dynamics simulations and by ^13^C nuclear magnetic resonance (NMR) spectroscopy, respectively [25,26]. The various physicochemical parameters of champagne (i.e., the liquid phase) and gas-phase CO_2_ (i.e., the bubbles), as determined at 20 °C, are reported in Table 1. These parameters were retrieved in our numerical procedures.

## 3. Numerical Methods and Set-Up

In this study, a CFD commercial code using the finite volume method was used for investigating the bubble-driven flow patterns found in a glass of champagne. In order to reproduce, as faithfully as possible, the flow patterns, it is necessary to consider the various physical processes at play, i.e., the nucleation of bubbles, the progressive release of carbon dioxide from the liquid phase, the interaction between ascending bubbles and the surrounding liquid phase, as well as the presence of a free surface between the liquid phase and the open atmosphere (in the 3D modeling only). As the problem involves several phases (champagne, CO_2_ bubbles and air), the use of a multiphase model was required. Firstly, 2D CFD simulations were carried out through an Eulerian–Lagrangian approach (with one Eulerian phase and one Lagrangian phase). In a second step, we used a 3D multiphase model combined with the volume-of-fluid (VOF) method (with two Eulerian phases and one Lagrangian phase) in an open channel flow configuration [27,28]. The VOF method was used to model the interaction between the liquid phase (champagne) and the gaseous phases (both ascending CO_2_ bubbles and open atmosphere). This interface tracking method is usually used to follow the position of the interface between the fluids [29,30,31,32]. In complement, we applied the open channel flow condition [33,34] to simulate the presence of a free surface (i.e., the air/champagne interface). Because the CFD code cannot model the upward dynamics of CO_2_ bubbles, the discrete phase model (DPM) was used as a very efficient way to follow bubbles in a Lagrangian reference frame [35]. In a Lagrangian formulation, the trajectory of each ascending bubble is calculated individually at each time-step of the simulation.

### 3.1. Liquid-Phase Governing Equations

The liquid phase flow was considered as being governed by both the continuity and momentum conservation equations for laminar flows. The equations for the continuous phase of multiphase flows are derived from the Navier–Stokes equations [21]. Given the presence of dispersed particles within the liquid phase (i.e., the CO_2_ ascending bubbles), a volume averaging method was used to develop a set of partial differential equations that describes the conservation of mass and momentum. The conservation equations can be formulated as a volume-average equation, as follows.

*Continuity Equation for the liquid phase*:

$$\frac{\partial \varepsilon_{l}}{\partial t}+\nabla \cdot \left(\varepsilon_{l}V\right)=0$$

*Momentum Equation for the liquid phase*:

$$\partial_{l}\frac{\partial \left(\varepsilon_{l}V\right)}{\partial t}+\rho_{l}\nabla \cdot \left(\varepsilon_{l}VV\right)=-\nabla p+\nabla \cdot \left(\varepsilon_{l}\tau \right)+\varepsilon_{l}\rho_{l}g+F_{bf}$$

with $\varepsilon_{l}$ being the liquid holdup, V being the liquid velocity vector, $\rho_{l}$ being the liquid density, p being the scalar pressure, τ being the viscous stress tensor, ***g*** being the gravitational field and $F_{bf}$ being the forces that result from the interaction between each bubble and the liquid phase.

### 3.2. Discrete Phase Modeling

The CFD code predicts the trajectory of a discrete-phase particle (i.e., the CO_2_ bubble in the present case) by integrating the balance of forces, which is written in a Lagrangian reference frame [28]. This force balance can be written (for the vertical direction in Cartesian coordinates) according to the following relationship:

$$\frac{du_{p}}{dt}=F_{D}\left(u_{l}-u_{p}\right)+\frac{g_{z}\left(\rho -\rho_{l}\right)}{\rho }+F_{z}$$

with $u_{p}$ being the bubble velocity, $u_{l}$ being the fluid phase velocity, ρ being the bubble density, $\rho_{l}$ being the liquid density, *g_z_* being the gravity acceleration (operating in the *z* direction in Cartesian coordinates), $F_{D}\left(u_{l}-u\right)$ being the drag force per unit particle mass and with the additional force $F_{z}$ corresponding to the “virtual mass” force required to accelerate the fluid surrounding the bubble.

Until now, and to the best of our knowledge, the nucleation models implemented in commercial CFD codes do not allow us to correctly model the specificity of the bubble nucleation and rise observed in carbonated beverages in general and in Champagne wines in particular [36]. Actually, the bubble nucleation rates found in champagne glasses depend on the level of dissolved CO_2_ found in the liquid phase (i.e., the supersaturation ratio of CO_2_). Moreover, CO_2_ bubbles formed through heterogeneous nucleation on the bottom of the laser-etched glass grow in size as they rise toward the champagne surface because dissolved CO_2_ diffuses from the liquid phase toward gas-phase CO_2_ through the bubble interface [37]. The growth rate of ascending bubbles therefore strongly depends on several parameters of the liquid phase, including the level of dissolved CO_2_, the diffusion coefficient of CO_2_ and the liquid-phase viscosity, among many others, as already described by several authors working with champagne and beer [37,38,39]. More details can be found about effervescence and key parameters governing bubble nucleation and rise in champagne glasses in the review by Liger-Belair [37].

**Figure 4:**
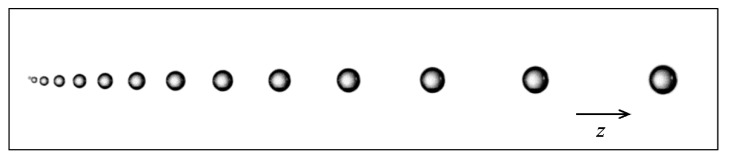
Train of successive spherical bubbles released with clockwork regularity from a specific nucleation site found at the bottom of a glass filled with champagne. Bubbles are seen growing in size and rising in line. User-defined functions (UDFs) implemented in the CFD code were based on the equations governing the dynamics of such spherical bubbles rising and growing in size in a solution supersaturated with dissolved CO_2_, as described in [21].

Based on the equations governing the dynamics of bubbles nucleating, rising and growing in size in a solution supersaturated with dissolved CO_2_, such as champagne, sparkling wines and beers, user-defined functions (UDFs) were implemented in the CFD code. UDFs were proposed to best model the frequency of bubble formation at the bottom of the glass, the bubble growth during ascent, the drag force exerted by the liquid phase on bubbles and the subsequent continuous acceleration experienced by ascending bubbles. More details about each and every UDF implemented in the CFD code can be found in the articles by Beaumont et al. [19,20,21]. It is worth noting that bubbles are injected into the fluid domain (at the bottom of the glass) with a trajectory that faithfully reproduces the dynamics of a single ascending and growing bubble [37]. Actually, bubbles rising at small to intermediate Reynolds numbers rise in line, in a straight-line path and remain nearly spherical, as described in previous works [38,39,40]. A photograph showing a train of successive bubbles rising and growing in line in a champagne glass is displayed in Figure 4.

In our CFD simulations, bubbles leave the injection points periodically, rise in line, grow in size and accelerate according to our previous experimental observations of ascending bubbles in champagne and sparkling wines, described in minute detail in [37]. Nevertheless, it is important to note that the various UDFs implemented in the CFD code were based on the equations governing the dynamics of single spherical bubbles, rising and growing in size in a solution supersaturated with dissolved CO_2_. However, in fact, under standard champagne tasting conditions, the myriad of ascending bubbles strongly interact with each other. Wake interactions between successive bubbles, bouncing or coalescence events [41,42,43] likely to occur in the central bubbly flow were not taken into account in our CFD simulations. The growth and rise of interacting bubbles require much more complex models, which go well beyond the scope of this paper. Our simplified procedure is therefore considered as a first step toward a more complex numerical study including the interactions between multiple bubbles, as for example, in the recent article by Lai et al. [44] and the references therein.

### 3.3. Boundary Conditions

The boundary conditions used in this work are the following:

Particles (i.e., the CO_2_ bubbles) were injected at the bottom of the glass and tracked with the fluid flow time step. The initial CO_2_ bubble diameter was equal to 5 × 10^−5^ m, in accordance with previous observations conducted through high-speed video imaging [37].

Particles were lost from the calculation at the free surface. Interactions between successive bubbles were neglected.

No-slip wall conditions were applied for the glass wall.

A pressure outlet condition was used at the outflow.

Free surface level was specified in open channel flow boundary conditions (for the 3D simulations).

A geometric reconstruction scheme was applied to predict the free surface shape between the atmosphere and the liquid (for the 3D simulations).

Numerical simulations were performed with ANSYS FLUENT^®^ 19.2 software, which is based on the finite volume approach. Ascending bubbles were subjected to low to moderate Reynolds numbers [40] and the flow was assumed to be laminar and governed by the finite volume equations. The convergence criteria were based on residuals whose values were monitored throughout the iterative calculation process. For all simulations carried out in this study, convergence of the results was achieved with residuals lower than 10^−5^. It is worth noting that the VOF method (needed in the 3D multiphase simulations) used a variable volume fraction to capture the air–champagne interface. To accurately represent the presence of the open atmosphere above the champagne surface, a buffer zone of air was defined as a 2 mm thick layer above the champagne surface. This buffer zone was the second Eulerian phase, the first Eulerian phase being the liquid phase (i.e., the champagne itself).

## 4. Results and Discussion

The ability of the CFD code to reproduce the dynamics of the self-organized ascending bubble-driven flow patterns found in a champagne glass was examined in light of previous sets of experimental data collected through laser tomography and PIV techniques. The two following paragraphs present a comparison between the flow patterns observed experimentally through PIV in the champagne glass described above, and the subsequent flow patterns resulting from the numerical 2D and 3D models, respectively.

### 4.1. The Two-Dimensional (2D) Model

**Figure 5:**
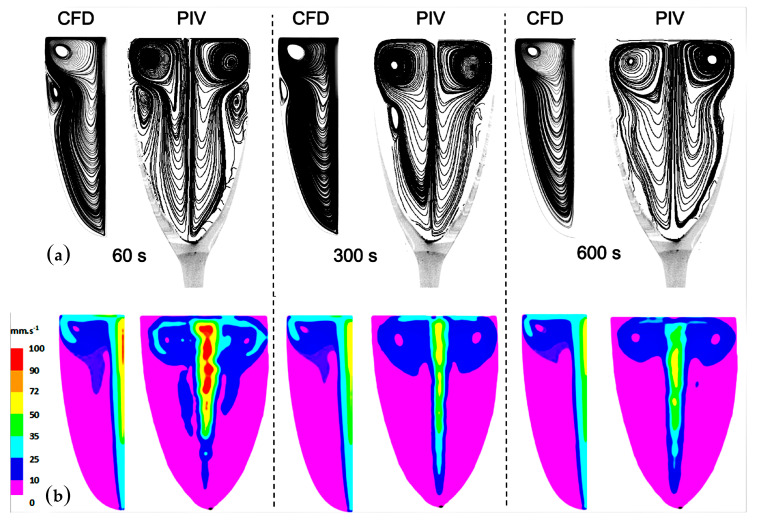
Comparison between the CFD and particle image velocimetry (PIV) streamline patterns as time proceeds (respectively 60 s, 300 s and 600 s after champagne was poured into the glass) (**a**); comparison between the CFD and PIV velocity fields as time proceeds (respectively 60 s, 300 s and 600 s after champagne was poured into the glass) (**b**).

The comparison between the dynamics of the self-organized ascending bubble-driven flow patterns, as determined from the 2D calculations, with previous experimental results obtained through 2D PIV techniques [15,16,18] is displayed in Figure 5. The comparison between the experimental and numerical streamlines shows a satisfactory agreement, especially regarding the flow topology and its evolution over time in Figure 5a. The 2D CFD model provides a realistic approach to the overall flow structure and its evolution as the concentration of dissolved CO_2_ champagne progressively decreases over time. Actually, the 2D CFD model numerically reproduces the global shape of the flow patterns observed through PIV and its evolution with time, with a main vortex ring that occupies nearly all of the computational domain and a second one of a much smaller size close to the glass wall. The second, smaller vortex, situated about 2 cm below the champagne surface, counter-rotates with respect to the main vortex, and progressively decreases in size before finally vanishing over time. Nevertheless, it is worth noting that small topological differences can be found between the numerical and experimental data for the flow patterns found immediately below the air/champagne interface, especially for the vortex core location.

After 10 min, we can see that the second vortex ring has disappeared from the CFD simulation, while a very slight inflection of the streamlines is still visible, both with the PIV and the CFD simulation. This secondary vortex ring maybe still exists 10 min after champagne is poured into the glass, but its vorticity and size are so small that it no longer appears in the streamline patterns. Nevertheless, it seems clear to us that CFD can predict the overall behavior of the flow patterns, by detecting the small secondary vortex and by showing its progressive decrease in size as time proceeds. Moreover, from the velocity contour maps resulting from the ascending bubbly flow shown in Figure 5b, it can be stated that the 2D CFD model is also in very good agreement with the experimental velocity field as determined through PIV. The highest fluid velocities were found in the wake of the central bubbly flow. In the wake of the central bubbly flow, the liquid phase reaches a maximum velocity in the order of 10 cm s^−1^ (in the zone marked with the red color in Figure 5b). Driven upward by the central bubbly flow, the liquid phase radially migrates close to the air/champagne interface, before plunging back downward into the liquid bulk close to the edge of the glass, thus initiating a swirling flow clearly visible in the streamline patterns displayed in Figure 5a. Sixty seconds after pouring champagne into the glass, the radial (subsurface) flow velocity ranges between 2.5 and 3.5 cm s^−1^ (in the light blue zone in Figure 5b). Moreover, both the experimental and CFD velocity contour maps displayed in Figure 5b show that the velocities of the liquid phase progressively decrease with time, which is particularly visible in the central area of the glass.

It can therefore be concluded that, despite some topological discrepancies, 2D numerical simulation allows a satisfactory approach to the self-organized bubble-driven flow patterns found in real laser-etched champagne glasses. These slight topological discrepancies may arise due to the boundary conditions at the air/champagne interface but also because of the non-perfectly axisymmetric conditions found in real champagne glasses.

### 4.2. The Three-Dimensional (3D) Model

**Figure 6:**
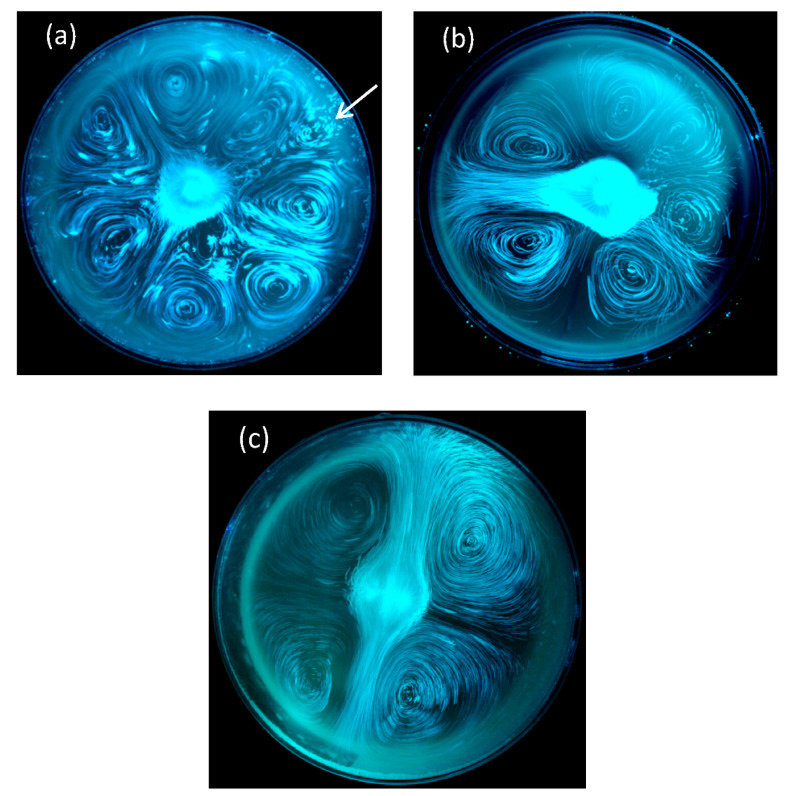
Laser tomography combined with long exposure photography showing fluid in motion at the free surface of a coupe filled with 100 mL of champagne. In frame (**a**), a highly unstable eight-cell regime is evidenced (soon after pouring the champagne), usually followed by a poorly stable six-cell regime (**b**) and then by a highly stable and long lasting four-cell regime, several minutes after pouring champagne into the coupe, where four counter-rotating cells self-organize at the air/champagne interface (**c**) [45].

In recent years, laser tomography and PIV techniques have been used to highlight the self-organized ascending bubble-driven flow patterns in various laser-etched champagne glasses. Previous experiments revealed that the overall flow patterns found in glasses mainly depends on glass shape combined with the intensity of the central bubbly flow (and therefore on the level of dissolved CO_2_ found in champagne) [13,14,15,16,17,18]. Moreover, and most interestingly, in a standard laser-etched coupe filled with champagne, self-organized and counter-rotating two-dimensional convective cells were also unveiled at the air/champagne interface [45]. Various regimes were evidenced, from a highly unstable eight-cell regime, to a very stable four-cell regime [45], as shown in Figure 6. There are indeed eight cells counter-rotating close to each other in Figure 6a (with seven big cells and a much smaller one marked with a white arrow). From the topological point of view, the four-cell regime displayed in Figure 6c looks strikingly like the so-called steady streaming flow, resulting from the action of an oscillatory cylinder in the main body of a fluid or in thin boundary layers [46,47,48].

**Figure 7:**
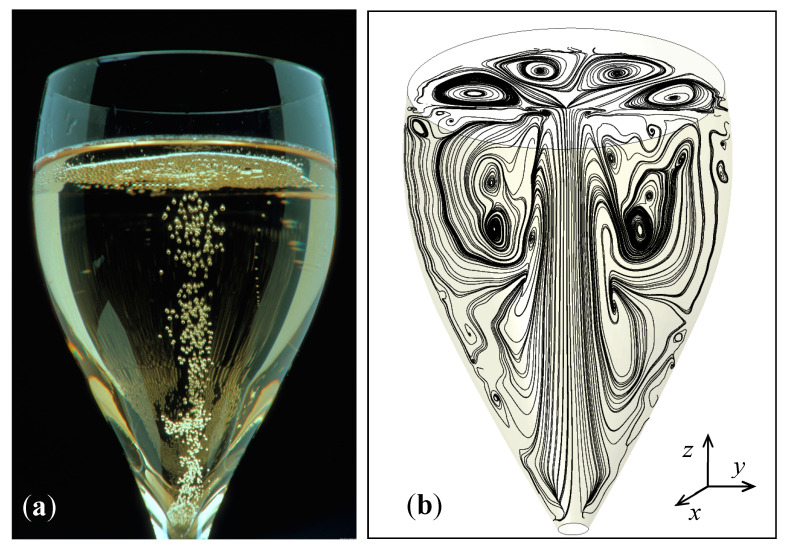
The central bubbly flow ascending in a laser-etched glass filled with 100 mL of champagne (**a**) has the ability to set up a network of various convective cells, invisible to the naked eye, but revealed through the 3D CFD model, in the plane of symmetry of the glass and at the air/champagne interface (**b**). (Photograph by Alain Cornu/Collection CIVC Authors have the permission to reuse this photography).

At the time we made these observations, we suggested that the 2D convective cells found at the air/champagne interface are interdependent with the ascending central bubbly flow and with the subsequent overall flow patterns found in the liquid bulk below the champagne surface [45]. A 3D CFD model was therefore needed in order to provide evidence of the three-dimensional nature of these instabilities. Figure 7a shows the column of ascending bubbles found in a laser-etched glass filled with 100 mL of champagne, and the subsequent ascending bubble-driven flow patterns, as determined through the 3D CFD model in Figure 7b, with the key parameters required by the UDFs implemented in the code, such as being able to numerically reproduce the highly unstable eight-cell regime found at the air/champagne interface. In Figure 7b, the streamlines are represented in two mutually perpendicular cross-sectional planes (x-y and y-z planes). Post-processing of the 3D simulations therefore revealed a highly complex network of various convective cells driven by the central bubbly flow (in the champagne bulk and at the air/champagne interface), thus confirming the close interplay between the flow patterns at the air/champagne interface and the flow patterns found in the subsurface fluid layers.

**Figure 8:**
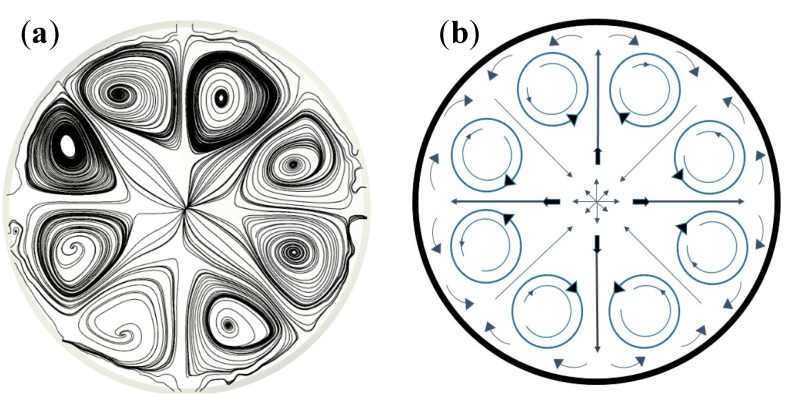
Close-up view of the 2D eight-cell regime found at the air/champagne interface, as determined through the 3D CFD model (**a**) and a subsequent layout showing how the fluid self-organizes at the air/liquid interface (**b**).

A close-up view of the self-organized 2D eight-cell regime found at the air/champagne interface, as determined through the 3D CFD model, is displayed in Figure 8a. A schematic layout is displayed in Figure 8b, which shows eight identical counter-rotating cells confined within a network of eight branches of fluids alternatively diverging and converging with regard to the center of the glass. In the center of the glass, the emerging bubbly flow pushes the fluid radially across the air/champagne interface, where the 2D flows split and self-organize along several preferential directions. The 2D eight-cell regime found at the air/champagne interface is therefore characterized by four branches of fluid diverging from the center of the glass, and four others converging toward the center of the glass, as detailed in Figure 8b.

It should be noted that perfectly symmetrical conditions can indeed be reached with a CFD simulation (as shown in Figure 8a, for example). Nevertheless, under real experimental conditions, the ascending bubbly flow resulting from the laser-etched glass is never perfectly centered, and tiny undesired bubble trains can also randomly nucleate on the glass wall, thus slightly affecting the overall symmetry of the 2D convective cells found at the air/champagne interface, as shown in Figure 6. The eight 2D counter-rotating cells, as determined through the 3D CFD model, are therefore clearly confined within the eight branches of fluid, at 45° angles to each other, and cross themselves in the center of the glass.

**Figure 9:**
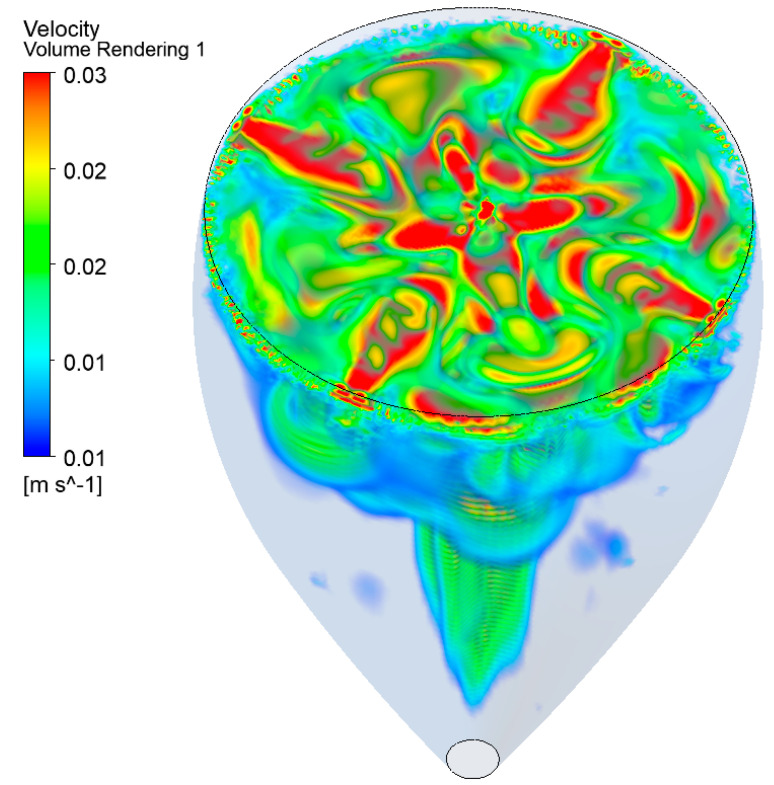
Volume velocity field resulting from the 3D simulations, with the key parameters required by the UDF, such as being able to numerically reproduce the highly unstable eight-cell regime found at the air/champagne interface.

The volume velocity field resulting from the 3D simulations, corresponding to the streamlines shown in Figure 7b and Figure 8a, is displayed in Figure 9. At the air/champagne interface, the interplay between the four branches of fluid diverging from the center of the glass and the four others converging is clearly visible. Actually, the highest velocity zones can clearly be identified along the eight branches of fluid crossing in the center of the glass.

It is worth noting that the mass flow rate of the ascending central bubble-driven flow is the key parameter conditioning the overall flow structure (both in the champagne bulk, and at the air/champagne interface, which evolves with a continuously decreasing number of counter-rotating convection cells). As time proceeds, when the central bubbly flow decreases (in bubbling frequency, bubble size and bubble velocity), the 2D flow pattern reorganizes itself in a highly stable and long lasting four-cell regime, as shown in Figure 6c. Moreover, from the experimental point of view, the diameter of the glass, as well as the level of champagne found in the glass, are also both strongly suspected to be key parameters conditioning the 2D flow pattern found at the air/champagne interface. Further 3D CFD simulations (and also further classical PIV experiments) are thus still needed to more deeply explore each and every parameter responsible for the transitions between the various flow regimes likely to be observed at the air/champagne interface.

## 5. Conclusions and Prospects

Computational fluid dynamics (CFD) was used as a very efficient tool for investigating the self-organized ascending bubble-driven flow patterns found in laser-etched champagne glasses. Based on the Eulerian–Lagrangian approach, CFD modeling was used in order to examine how a column of ascending bubbles nucleated at the bottom of a laser-etched champagne glass can drive a complex network of counter-rotating convective cells, both in the champagne bulk and at the air/champagne interface. Results from 2D CFD axisymmetric simulations were compared with a previous set of experimental data conducted through particle image velocimetry (PIV). Despite slight topological discrepancies, 2D numerical simulation allowed a satisfactory approach to the self-organized bubble-driven flow patterns found in real laser-etched champagne glasses. A 3D CFD model was also developed, by using the conventional volume-of-fluid (VOF) multiphase method, in order to explore the connections between the ascending bubble-driven flow patterns found in the champagne bulk and the subsequent 2D self-organized flow patterns found at the air/champagne interface. In complete accordance with several experimental observations conducted through laser tomography and PIV techniques, CFD revealed a very complex flow composed of surface eddies interacting with a toroidal flow that develops around the ascending bubble column. Combined with conventional flow visualization techniques, CFD proved to be a useful tool in order to explore and study complex two-phase flow patterns, such as those surprisingly found in a single glass of champagne.

Including the multiple bubble interactions in our CFD procedures could also be the purpose of future works in order to improve this first approach. In a near future, CFD could thus be considered a valuable tool capable of providing new insights into the three-dimensional swirling structure of ascending bubble-driven flow patterns found in every glass filled with a sparkling beverage. The predictive capabilities of CFD pave the way for the creation of an effective numerical tool dedicated to glassmakers and designers. The ultimate goal of our approach is to quickly and cost effectively analyze the influence of glass shape on the subsequent ascending bubble-driven flow patterns influencing, in turn, the release of dissolved CO_2_, as well as the evaporation of VOCs in the headspace above glasses.

## Figures and Tables

**Table 1:** Physicochemical parameters of champagne and gas-phase CO_2_ (at 20 °C).

	Champagne	Gas-Phase CO_2_
Density *ρ* (kg m^−3^)	9.98 × 10^2^	1.79
Dynamic viscosity *η* (kg m^−1^ s^−1^)	1.56 × 10^−3^	1.37 × 10^−5^
Surface tension *γ* (mN m^−1^)	46.8	/
Dissolved CO_2_ concentration [CO_2_]_ini_ (g L^−1^)	7.4	/
CO_2_ diffusion coefficient *D* (m^2^ s^−1^)	≈1.4 × 10^−9^	/
